# Evaluation of (−)-epicatechin metabolites as recovery biomarker of dietary flavan-3-ol intake

**DOI:** 10.1038/s41598-019-49702-z

**Published:** 2019-09-11

**Authors:** Javier I. Ottaviani, Reedmond Fong, Jennifer Kimball, Jodi L. Ensunsa, Nicola Gray, Anna Vogiatzoglou, Abigail Britten, Debora Lucarelli, Robert Luben, Philip B. Grace, Deborah H. Mawson, Amy Tym, Antonia Wierzbicki, A. David Smith, Nicholas J. Wareham, Nita G. Forouhi, Kay-Tee Khaw, Hagen Schroeter, Gunter G. C. Kuhnle

**Affiliations:** 1 Mars, Inc., McLean, VA USA; 2 Department of Nutrition, UC Davis, Davis, CA USA; 3 Department of Food and Nutritional Sciences, University of Reading, Reading, UK; 4 School of Life Sciences, Coventry University, Coventry, UK; 5 MRC Epidemiology Unit, University of Cambridge, Cambridge, UK; 6 Department of Public Health and Primary Care, University of Cambridge, Cambridge, UK; 7 LGC, Newmarket Road, Fordham, UK; 8 OPTIMA Department of Pharmacology, University of Oxford, Oxford, UK

**Keywords:** Epidemiology, Risk factors

## Abstract

Data from dietary intervention studies suggest that intake of (−)-epicatechin mediates beneficial vascular effects in humans. However, population-based investigations are required to evaluate associations between habitual intake and health and these studies rely on accurate estimates of intake, which nutritional biomarkers can provide. Here, we evaluate a series of structurally related (−)-epicatechin metabolites (SREM), particularly (−)-epicatechin-3′-glucuronide, (−)-epicatechin-3′-sulfate and 3′-O-methyl-(−)-epicatechin-5-sulfate (SREM_B_), as flavan-3-ol and (−)-epicatechin intake. SREM_B_ in urine proved to be a specific indicator of (−)-epicatechin intake, showing also a strong correlation with the amount of (−)-epicatechin ingested (R^2^: 0.86 (95% CI 0.8l; 0.92). The median recovery of (−)-epicatechin as SREM_B_ in 24 h urine was 10% (IQR 7–13%) and we found SREM_B_ in the majority of participants of EPIC Norfolk (83% of 24,341) with a mean concentration of 2.4 ± 3.2 µmol/L. Our results show that SREM_B_ are suitable as biomarker of (−)-epicatechin intake. According to evaluation criteria from IARC and the Institute of Medicine, the results obtained support use of SREM_B_ as a recovery biomarker to estimate actual intake of (−)-epicatechin.

## Introduction

There is considerable evidence suggesting that flavanols can be beneficial for cardiovascular disease prevention and health maintenance, although current data are still insufficient to provide general dietary guidance^1,2^. Recent data have shown that the monomer (−)-epicatechin, but not oligomeric and polymeric procyanidins, are responsible for improvements in vascular function^3^. While the effect of (−)-epicatechin on health can be investigated in clinical dietary intervention studies, only epidemiological investigations can provide population-based information about the long-term effect of (−)-epicatechin as part of the habitual diet. However, such investigations rely on an accurate estimate of intake, and this is not possible with the methods commonly used today. Reasons lie in the significant limitation of self-reported dietary data as well as in the large variability of the (−)-epicatechin content of foods (Table 1). Alternatives to the latter approach emerge in the use of nutritional biomarkers of intake, which reflect actual intake and do not rely on self-reported food intakes and food composition data^4,5^. While we have recently evaluated a nutritional biomarker of flavan-3-ol intake^6^, there are currently no established biomarkers to specifically assess the intake of (−)-epicatechin.

**Table 1:** Ranges of (−)-epicatechin content (mg/100 g or mg/100 mL) of different foods commonly consumed (data from Phenol Explorer 3.0^37^).

Food	Content [mean ± SD, range]
Apple (Cider), peeled	28.7 ± 26.5 (0.0–141.0)
Apple (Dessert), peeled	6.7 ± 4.5 (0.0–19.8)
Apricot, raw	3.5 ± 4.3 (0.0–6.1)
Blackberry, raw	11.5 ± 10.9 (2.7–18.1)
Chocolate, dark	70.4 ± 29.5 (32.7–125.0)
European cranberry	4.2 ± 0.0 (4.2–4.2)
Grape (Black)	5.2 ± 5.6 (0.7–8.6)
Nectarine, peeled	3.0 ± 1.1 (1.3–5.6)
Peach, peeled	8.0 ± 4.2 (0.7–16.5)
Pear, whole	3.8 ± 2.7 (0.2–7.5)
Red raspberry, raw	5.0 ± 3.8 (0.3–8.3)
Sweet cherry, raw	7.8 ± 2.9 (5.5–9.5)
Tea (Black), infusion	3.9 ± 4.3 (0.0–16.8)
Tea (Green), infusion	7.9 ± 13.7 (0.0–73.9)
Tea (Oolong), infusion	2.7 ± 3.8 (0.0–13.2)
Wine (Red)	3.8 ± 3.2 (0.0–16.5)

Nutritional biomarkers for phenolic compounds such as (−)-epicatechin, are often based on the concentration of their metabolites in blood or urine^7^. However, a careful evaluation of phenolic metabolites is important to assess their suitability and to identify their strengths and limitations as biomarkers of intake. Previously, we used the evaluation process proposed by IARC (International Agency for Research on Cancer) and the IOM (Institute of Medicine)^6^ to evaluate 5-(3′,4′-dihydroxyphenyl)-γ-valerolactone (gVL) and its metabolites (gVLM) as nutritional biomarker of flavan-3-ol intake^6^. These criteria include: (i) demonstration of accuracy, precision and reliability of the analytical method to quantify the candidate biomarker; (ii) demonstration and characterization of the relationship between intake and candidate biomarker levels in urine, including characterizing specificity and intra-individual variability; and (iii) demonstration of the applicability of the candidate biomarker to estimate intake in large cohort studies. Here, we apply the same criteria to evaluate three of the most abundant structurally related (−)-epicatechin metabolites (SREM), including (−)-epicatechin-3′-glucuronide (E3′G), (−)-epicatechin-3′-sulfate (E3′S) and 3′-*O*-methyl-(−)-epicatechin-5-sulfate (3MeE5S), as biomarker of (−)-epicatechin intake and to estimate (−)-epicatechin intake in large-scale cohort studies using this biomarker.

## Methods

### Analytical method – epidemiological study

Analysis was performed using ultra high-performance liquid chromatography coupled to tandem mass spectrometry (UPLC-MS/MS; Waters Acquity BSM, coupled with an Applied Biosystems API 4000; see Supplemental Fig. 1 for a typical chromatogram). *De novo* synthesized authentic standards^8,9^ of three human structurally related epicatechin metabolites (SERMs) considered in this study ((−)-epicatechin-3′-glucuronide (E3′G), (−)-epicatechin-3′-sulfate (E3′S) and 3′-O-methyl-(−)-epicatechin-5-sulfate (3Me5S)), as well as stable isotope labelled internal standards (ISTD, D_2_/D_3_-epicatechin-3′-β-D-glucuronide, 50:50 mix) were obtained from Analyticon (Analyticon Technologies AG, Lichtenfels, Germany; Supplemental Fig. 2 for structures). Purity was assessed by LC-UV and LC-MS, and stock solutions were adjusted accordingly. Stock solutions were prepared in ethanol:water (70:30, v/v) and stored at −80 °C.

Flavan-3-ol metabolite-free spot urine was obtained from human volunteers on a low-flavan-3-ol diet, excluding in particular tea, pome fruits and berries from their diet. This study was approved by the University of Reading Research Ethics Committee. All participants gave their written informed consent to participate and all experiments were performed in accordance with relevant University guidelines and regulations and followed GCP (good clinical practice).

Urine samples (60 µL) and internal standard solutions (1.0 µM ISTD, 60 µL) were combined, filtered (Impact Protein Precipitation filter plate, Phenomenex, Macclesfield, UK) by centrifugation (500 × g) for 2 minutes at room temperature and stored at −20 °C until analysis. Sample preparation was automated for the analysis of EPIC (European Prospective Investigation into Cancer) Norfolk cohort urine samples using a Hamilton Star robot (Hamilton, Bonaduz, Switzerland).

Samples were separated by liquid chromatography (Acquity, Waters, Elstree, UK) using a C18 column (Kinetex C18 100 × 2.1 mm, 1.7 µm, with 0.5 µm Krudcatcher, Phenomenex, Macclesfield, UK). Gradient elution was performed at a flow rate of 0.5 mL/min using 0.1% formic acid in water as mobile phase A and acetonitrile/methanol (9:1, v/v) as mobile phase B. The initial starting conditions were 95% A, which were held for 2 min, decreasing to 85% A at 3.5 min, 79.6% at 6.0 minutes 5% A at 6.1 minutes and held until 7.0 minutes to wash the column before returning to 95% A at 7.1 minutes for re-equilibration until 8 minutes. The column temperature was maintained at 25 °C and the injection volume was 10 µL.

Compounds were detected using an Applied Biosystems Sciex API 4000 instrument (Warrington, UK) equipped with Turbo Ion Spray probe operating in negative ion mode using the parameters shown in Table 2. The spray voltage was −4500 V and the source temperature was 600 °C. The dwell time was 20 ms for each transition.

**Table 2:** LC-MS parameters for analytes and internal standards considered in method including precursor and product ions (m/z) and typical retention times.

Analyte	Precursor ion (m/z)	Product ion (m/z)	Retention time (min)
(−)-epicatechin-3′-glucuronide (E3′G)	464.8	289.1	4.2
(−)-epicatechin-3′-sulfate (E3′S)	369.1	289.1	4.3
3′-O-methyl-(−)-epicatechin-5-sulfate (3Me5S)	382.9	303.1	4.6
D_2_-epicatechin-3′-β-D-glucuronide (D2-E3′G)	466.6	291.1	4.1
D_3_-epicatechin-3′-β-D-glucuronide (D3-E3′G)	468.3	292.1	4.1

Samples quantified using calibration standards prepared in flavan-3-ol metabolite-free urine samples (standard concentrations [µM] were prepared at 0.1, 0.25, 1, 2, 3, 4, 5). E3′G, E3′S and 3Me5S were quantified using the peak area ratio of analyte and ISTD (D2/D3-E3′G, 50:50 (w/w), where the D3-E3′G precursor/product ions were used).

Each batch included two replicates of quality control (QC) samples with three different concentrations; low QC (0.3 µM), medium QC (2.5 µM) and high QC (3.8 µM). Usual acceptance criteria for each batch were that as least one QC at each concentration and four out of the six QCs were within 15% of the theoretical concentration.

The method was validated using flavan-3-ol metabolite-free urine, spiked with the authentic standards E3′G, E3′S and 3Me5S to assess stability, specificity, matrix effects, precision and accuracy. The matrix effect was assessed by spiking flavan-3-ol metabolite-free urines from different sources with known amounts of analyte and internal standard and comparing their peak-area-ratio ratios. Method performance was assessed using data from quality control samples in 255 batches analyzed.

### Human intervention studies

Three human intervention studies were conducted to investigate biomarker specificity, intake-response relationship and intra-individual variability. Details of the human intervention studies, with the exception of the investigation of intra-individual variability, have been described previously^6^. Briefly, the intervention consisted of fruit-flavoured non-dairy drinks that contained either specific flavan-3-ols (specificity study) or varying amounts of flavan-3-ols derived from cocoa (4 levels for the intake amount escalation study) and chocolate-flavored dairy drinks containing flavan-3-ols derived from cocoa (intra-variability study). The test materials were prepared freshly each day, and were matched regarding their macro- and micronutrient content and orosensory and physicochemical characteristics. All test materials were supplied by Mars, Incorporated (McLean, VA). A summary of the events taking during each study visit is summarized in Supplemental Fig. 3. These study protocols were approved by the Institutional Review Board of UC Davis. All participants gave their written informed consent to participate and all experiments were performed in accordance with local guidelines and regulations. These studies were registered as NCT03194620 and NCT03201822.

#### Study population

Healthy adults between 25 and 60 years of age (specificity study, Supplemental Fig. 4) and between 25 and 40 years of age (intake amount escalation study, Supplemental Fig. 5) were recruited by public advertisement in the city of Davis and surrounding areas (California, USA). Exclusion criteria were previously described as: body mass index (BMI) higher than 30 kg/m^2^, blood pressure (BP) higher than 140/90 mmHg, food allergies to test materials and a history of disease^6^. Throughout participation in the study (approximately, 8 weeks for the Specificity study, 4 weeks for the Intake escalation study and 2 weeks for the Intra-individual variability study), volunteers were asked to maintain their typical daily activities and diet. To control for potential dietary flavan-3-ol intake, volunteers were asked to follow a defined low-flavan-3-ol diet on the day prior to and during each study day, for a total of 2 days following a low-flavan-3-ol diet. All volunteers were instructed on how to follow a low-flavan-3-ol diet, receiving foods containing low or negligible amounts of flavanols including the dinner for the night previous to the study day. Volunteers were required to fast overnight (12 h water, *ad libitum*) before each study day.

#### Specificity study

In order to investigate the specificity of SREM_B_ as nutritional biomarker of flavan-3-ol intake, we performed a randomized, double-masked and 8×-crossover dietary intervention study with different possible precursors of structurally related (−)-epicatechin metabolites in healthy male adults (n = 12). Details of this study have been reported previously^6^. The flavan-3-ols tested were: (i) (–)-epicatechin, (ii) (−)-epigallocatechin, (iii) (−)-epicatechin-3-O-gallate, (iv) (−)-epigallocatechin-3-O-gallate (all compounds isolated from green tea, purity >99% and food grade), (v) a 1:1:1: mixture of theaflavin-3-O-gallate, theaflavin-3′-O-gallate and theaflavin-3,3′-O-digallate, (vi) thearubigins (all isolated from black tea, purity >95% and food grade) and (vii) procyanidin B-2 ((−)-epicatechin-(4α4β)-(−)-epicatechin; isolated from cocoa, purity 91%). One additional visit included the consumption of the test material without any flavan-3-ol added (control day). The amount of flavan-3-ols consumed per volunteer per study visit was equivalent to 34.8 mg or 120 µmol, which is close to the mean intake amount of (–)-epicatechin consumed in the UK^10^. Test materials were consumed on a single occasion in the morning of the study visit. After intake, urine was collected over 24 h in two collection periods (from 0 h to 6 h post intake and 6 h to 24 h post intake), using a fresh container for each collection period with 20 mL of 2 M sodium acetate (pH = 4.5) and 2 mL of 0.5% (w/v) thymol in isopropanol as preservatives. Volunteers returned containers upon completion of sample collection, and urine was stored at −80 °C until analysis. Volunteers were randomized to receive the 8 different test materials using computer-generated lists of random numbers via the randomly permuted block method. Study visits were separated by approximately 7 days for most volunteers, but never less than 3 days. The allocation list was generated by a researcher not involved with the recruitment and allocation of participants. Participants, study nurses, and researchers assessing outcomes as well as researchers involved with the statistical analysis of data were masked to the specific nature of intervention. The initial recruitment started in August 2016, and the study was conducted from August 2016 to January 2017.

#### Intake escalation study

In order to investigate the association between flavan-3-ol intake and SREM_B_ excretion, we conducted a dietary intervention study where participants consumed different amounts of flavan-3-ol. Details of this study have been reported previously^6^. This was a randomized, double-masked study and followed a 4×-crossover design, in which healthy male adults (n = 14) consumed four different amounts of flavan-3-ols that ranged from 95 mg to 1424 mg, including 15 mg to 227 mg of (−)-epicatechin, which are amounts including and exceeding the range of intakes determined in the UK^10^. Test materials were consumed on a single occasion in the morning of the study visit. After intake, urine was collected over 24 h in 4 collection periods (from 0 h to 4 h post intake, 4 h to 8 h post intake, 8 h to 12 h post intake and 12 h to 24 h post intake), using a fresh container for each collection period with 20 mL of 2 M sodium acetate (pH = 4.5) and 2 mL of 0.5% (w/v) thymol in isopropanol as preservatives. Volunteers returned the container upon completion of sample collection, and urine was stored at −80 °C until analysis. Volunteers were randomized to receive the four different test materials using computer-generated lists of random numbers via the randomly permuted block method. Study visits were separated by approximately 7 days for most volunteers, but never fewer than 3 days. The allocation list was generated by a researcher not involved with the recruitment and allocation of participants. Participants, study nurses, and researchers assessing outcomes as well as researchers involved with the statistical analysis of data were masked to the intervention. The initial recruitment started in April 2013, and the study was conducted from April to May 2013.

#### Intra-individual variability study

In order to investigate the intra-individual variation of SREM_B_ excretion, we conducted a dietary intervention study where male and female participants consumed the same flavan-3-ol-containing drink on two different occasions separated by not less than 3 days (for study population see Supplemental Fig. 6). The amount of flavan-3-ols ingested was 542 mg, including 76 mg of (−)-epicatechin, which is well within the range of intakes determined in the UK^10^. Test materials were consumed on a single occasion in the morning of the study visit. After intake, urine was collected over 24 h in 2 collection periods (from 0 h to 6 h post intake and 6 h to 24 h post intake), using a fresh container for each collection period with 20 mL of 2 M sodium acetate (pH = 4.5) and 2 mL of 0.5% (w/v) thymol in isopropanol as preservatives. Volunteers returned the container upon completion of sample collection, and urine was stored at −80 °C until analysis. Study visits were separated by approximately 7 days for most volunteers, but never less than 3 days. The initial recruitment started in July 2018, and the study was conducted from August to September 2018.

#### Analytical method – human intervention studies

Urine samples from human intervention studies (50 µL) were diluted with 100 µL of 100 µM 3-methyl hippuric acid (internal standard) and analyzed by UPLC-MS within 24 h, using a method described previously^6^. Samples were separated on a Waters Acquity UPLC (Waters, Milford, MA, USA) with an autosampler cooled at 5 °C. The injection volume was 37.5 µL. Chromatography was carried out at 25 °C using a 100 × 2.1 mm Kinetex C18 1.7 µm, reversed phase column with a Krudcatcher (Phenomenex). The mobile phase, pumped at a flow rate of 0.5 mL/min, was (A) 5 mM ammonium formate (pH 2.9); and (B) acetonitrile/methanol (90:10, v/v). The gradient started with 5% B from 0–2 min, then ramped to 17.5% from 2–3.5 min, and then to 22.5% from 3.5–6 min. Detection was achieved with a Waters Micromass Quattro Premier spectrometer fitted with an electrospray interface (ESI; Waters). MS analysis was carried out in negative ionization mode by multiple reaction monitoring (MRM). Detection of (−)-epicatechin-3′-glucuronide, (−)-epicatechin-3′-sulfate, and 3′-O-methyl-(−)-epicatechin-5-sulfate utilized transitions from 464.5 m/z to 289 m/z, 368.8 m/z to 289 m/z, and 382.5 m/z to 302.5 m/z, respectively with dwell times of 0.05 s, 0.1 s, and 0.1 s; cone voltages of 25 V, 30 V, and 35 V; and collision energies of 18 V, 20 V, and 18 V respectively. Data processing was performed using Masslynx software (Waters). MS conditions were set by tuning with the corresponding compound. Source and desolvation temperature were set at 150 °C and 500 °C, respectively, while desolvation and cone gas were set at 900 L/h and 30 L/h, respectively. In both ionization modes, capillary, cone, and lens voltage were set at 4 kV, 30 V, and 1 V, respectively. The identification and quantification of the compounds were based on co-elution with authentic standards. The precision and accuracy of the method was better than 5% and 20%, respectively.

### Cohort studies

We have investigated the feasibility of SREM_B_ as nutritional biomarker of (−)-epicatechin in samples from two cohort studies, EPIC Norfolk and Challenge.

#### EPIC Norfolk

25,639 apparently healthy men and women between 40 and 75 years of age, living in and around Norwich, UK, were recruited between 1993 and 1997^11^. Spot urine samples were collected during the baseline health examination and stored at −20 °C; 24,341 spot urine samples were available for analysis. The study was approved by the Norwich District Health Authority Ethics Committee, and all participants gave signed informed consent.

#### Challenge study

158 cognitively normal elderly people ((77 women, 81 men) aged 60 to 91 years old) living in and around Oxford were recruited in 1997 as part of the OPTIMA (Oxford Project to Investigate Memory and Ageing) study^12^. 24 h urine samples were collected at baseline and stored at −70 °C; 145 samples were available for analysis. The Central Oxford Research Ethics Committee granted ethical approval. Informed consent was obtained from all participants for all testing.

### Statistical analyses

Associations between biomarker and intake were assessed with linear regression models using R 3.5.0^13^ with the *boot*^14^, *ggplot2*^15^ and *rms*^16^ packages. Differences between epicatechin precursors where investigated using one-way ANOVA with Tukey’s Honest Significant Difference method^17^.

## Results

### Analytical method validation and performance

The analysis of nutritional biomarker requires robust and validated analytical methods. We have included the sum of three SREMs ((−)-epicatechin-3′-glucuronide (E3′G), (−)-epicatechin-3′-sulfate (E3′S) and 3′-O-methyl-(−)-epicatechin-5-sulfate (MeE5S), SREM_B_) in an LC-MS method developed for the analysis of gVLM (5-(3′,4′-dihydroxyphenyl)-γ-valerolactone-3′/4′-O-glucuronide (gVL34G) and 5-(3′,4′-dihydroxyphenyl)-γ-valerolactone-3′/4′-sulfate (gVL34S)) as biomarker of flavan-3-ol intake, as this method can be automated and is suitable for high-throughput analysis^6^. The precision and accuracy of the method was well within the usual GLP criteria of 15% and better than 10% for most compounds. The estimated limit of detection (signal-to-noise ratio better than 5-to-1) was at least 0.1 µM for all compounds. Where matrix effects were evident, these were adequately compensated for by the use of an isotope-labelled internal standard. All compounds were tested for their stability in urine under different conditions, including at high temperature (6 h at 37 °C, 24 h at room temperature) and following four freeze-thaw cycles (−80 °C), and the extracted samples were tested for 72 h at 5 °C and seven days at −20 °C. All compounds were stable under these conditions and showed no indication of deterioration and we did not observe any batch effect or long-term drift of results. Table 3 shows long term precision and accuracy data for the three (−)-epicatechin metabolites (SREM_B_).

**Table 3:** Long term precision (%CV) and accuracy (%RE, difference of mean calculated concentration and nominal concentration, standardized by nominal concentration) data for the three SREMs used as biomarker (SREM_B_), based on 575 samples (except (−)-epicatechin-3′-sulfate high QC for which 370 samples only were available).

	Low QC 0.3 µM			Medium QC 2.5 µM			High QC 3.8 µM		
	Mean (SD)	%CV	%RE	Mean (SD)	%CV	%RE	Mean (SD)	%CV	%RE
(−)-epicatechin-3′-glucuronide	0.32 (0.03)	10.0	6.4	2.6 (0.3)	9.4	4.5	3.9 (0.3)	7.5	1.9
(−)-epicatechin-3′-sulfate	0.32 (0.03)	9.3	5.4	2.6 (0.2)	8.5	2.7	3.8 (0.3)	7.7	1.1
3′-O-methyl-(-)-epicatechin-5-sulfate	0.32 (0.03)	10.4	6.4	2.6 (0.2)	8.8	4.1	3.9 (0.3)	8.6	1.4

**Figure 1:**
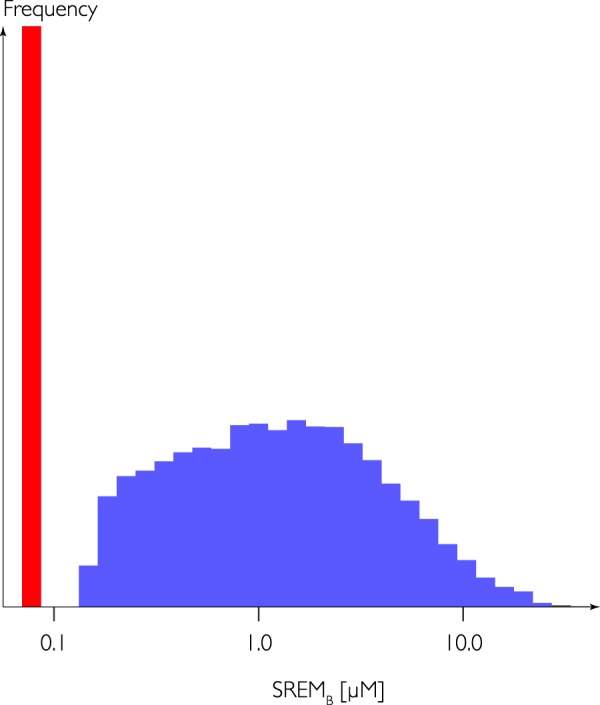
Histogram of urinary SREM_B_ concentration in spot urines of 24,341 participants of EPIC Norfolk. The lower limit of quantification was 0.1 µM.

In an analysis of spot urines collected from 24,341 participants of EPIC Norfolk, biomarker concentrations above the limit of quantification (0.1 µmol/L) were found in 20,277 participants (83%). Figure 1 shows the distribution of SREM_B_ concentrations, which was skewed to the left with a median (IQR) of 1.2 (0.5–2.9) µmol/L and a mean (SD) of 2.4 (3.2) µmol/L.

### Specificity of SREM_B_ as biomarker of flavan-3-ol intake

**Figure 2:**
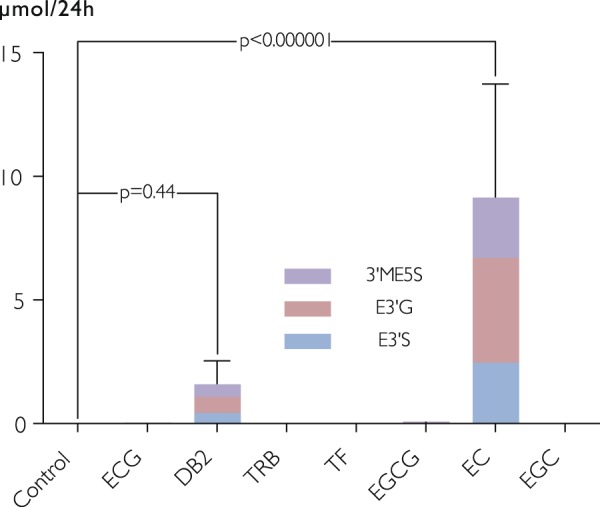
Urinary excretion of structurally related (−)-epicatechin metabolites (SREM_B_) following the consumption of different flavan-3-ols in male adults (n = 12). ECG, (−)-epicatechin-3-*O*-gallate; DB2, procyanidin dimer B2; TRB: thearubigins; TF: theaflavins; EGCG, (−)-epigallocatechin-3-O-gallate; EC, (−)-epigallocatechin; EGC, (−)-epigallocatechin. Differences in excretion were compared using a one-way ANOVA and a pair-wise comparison with Tukey’s Honest Significant Difference method^17^.

To assess potential precursors of SREMs, we investigated SREM_B_ formation following the consumption of different flavan-3-ols, including monomeric flavan-3-ols, oligomeric (procyanidins) and galloylated flavan-3-ols in male adults (n = 12). Figure 2 shows that only the consumption of (−)-epicatechin resulted in statistically significant increase of SREM_B_ excreted in urine. While the intake of procyanidin dimer B2 did not statistically increase SREM_B_ excreted in urine (p = 0.4; one-way ANOVA), the contribution could be estimated as 2% of the amount of procyanidin dimer B2 ingested. The rest of the flavan-3-ols ingested, including (−)-epicatechin-3-*O*-galate, did not contribute to the presence of SREM_B_ excreted in urine.

### Relationship between flavan-3-ol intake and SREM_B_

**Figure 3:**
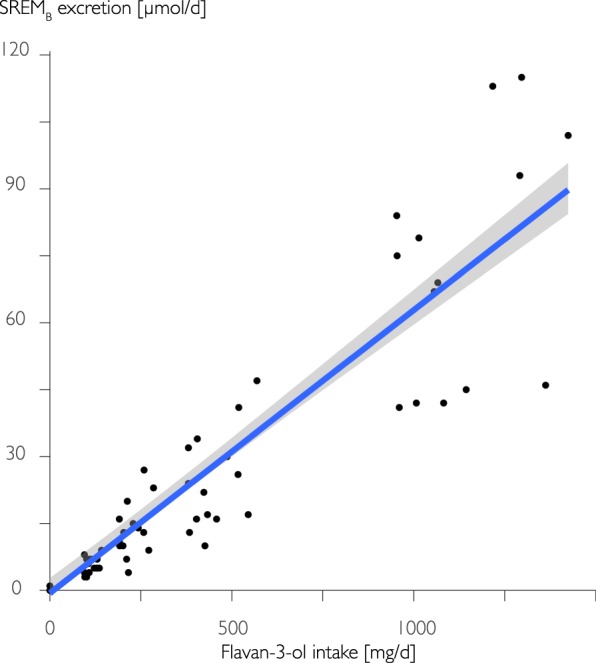
Association between flavan-3-ol intake and biomarker (SREM_B_) excretion in male adults (n = 14). The data show a strong correlation between intake and biomarker (R^2^: 0.86 (95% CI 0.8l; 0.92)).

A consistent and strong relationship between flavan-3-ol intake and SREM_B_ excretion in 24 h urine is crucial to establish its suitability as biomarker. We have therefore investigated this association with an intake-escalation study in healthy male adults (n = 14) with intakes of up to 1400 mg/d of flavan-3-ols (230 mg/d of (−)-epicatechin), which exceeds the range of likely dietary intake^10,18^. The results show a strong correlation between intake and SREM_B_ (R^2^: 0.86 (95% CI 0.8l; 0.92), Spearman’s rank correlation: ρ = 0.96; Pearson’s r = 0.93; Fig. 3). These results were only slightly attenuated when excluding high intakes above 750 mg/d (R^2^: 0.82 (95% CI 0.73; 0.88), Spearman’s rank correlation: ρ = 0.94; Pearson’s r = 0.90). We also investigated the extent of the relationship between (−)-epicatechin intake and SREM_B_. The results show that the relationship between dietary intake of (−)-epicatechin and SREM_B_ is β = 0.12 (95% CI 0.11; 0.12) with a median recovery of 10% (IQR 7–13%).

### Intra- and inter-individual variability

Intra- and inter-individual variability of the association between flavan-3-ol intake and SREM_B_ affect its suitability as biomarker of intake. We have therefore investigated intra- and inter-individual variability in men and women (n = 15, m/f: m/f) consuming 543 mg of flavan-3-ols (including 263 µmol (−) epicatechin) on two occasions. Mean (±SEM) SREM_B_ excretion at the first and second intervention was 46.2 ± 4.2 µmol and 42.8 ± 4.4 µmol respectively, with no statistically significant difference (paired t-test, p = 0.2). There was a strong correlation between SREM_B_ excretion at first and second visit (Pearson’s ρ: 0.83; R^2^ = 0.66). The intra-individual variability was 12 ± 2.5%, with only two participants having differences larger than 20%.

### Applicability of the candidate biomarker to estimate intake in free living individuals (Challenge study)

We analyzed 24 h urine (n = 158) collected in the context of the Challenge study. With a median recovery of 10% as found in the intake-escalation study, the estimated intake of (−)-epicatechin was of 14.0 ± 1 mg/d, ranging from 0 to 58 mg/d.

## Discussion

In this series of studies, we have investigated the suitability of three structurally related (−)-epicatechin metabolites ((−)-epicatechin-3′-glucuronide (E3′G), (−)-epicatechin-3′-sulfate (E3′S) and 3′-O-methyl-(−)-epicatechin-5-sulfate (MeE5S), SREM_B_) as nutritional biomarkers of dietary (−)-epicatechin intake using criteria by IARC^19^ and the Institute of Medicine^20^ as described previously^6^. Here, we discuss whether or not SREM_B_ is suitable as biomarker of flavan-3-ol intake:

### Measurement of SREM_B_ in urine using LC-MS

The application of any biomarker relies on the availability of accurate, precise and robust analytical methods, which ideally should be suitable for automation and high-throughput analysis to allow their application to large studies. We have developed an analytical method which is suitable for the rapid analysis of the two main types of flavan-3-ol metabolites, structurally related (−)-epicatechin metabolites (SREM) and metabolites of the microbial fission products of flavan-3-ols, γ-valerolactones^6^. This method allows us to determine a range of different metabolites in a short time, and therefore is suitable for the analysis of samples even from large studies. The accurate and precise analysis of these metabolites requires the availability of authentic standards, as other methods, relying for example on enzymatic deconjugation^21,22^ or the identification of metabolites using their tandem-MS fragmentation pattern and quantification as aglycone^23^, considerable bias. The measurement error introduced by these methods is dependent on metabolite and metabolite concentration, and therefore does not only affect absolute but also relative quantification. This makes such methods unsuitable to rank participants according to intake, as is common practice for many nutritional biomarkers. We have confirmed the performance of the method following generally accepted standards^24^, with precision and accuracy well below 15% for the entire concentration range. In combination with a run time of eight minutes and the suitability for robotic automation, this makes the method suitable for the high-throughput analysis of large-scale studies. The application of the method to two different cohort studies in the UK shows that the validation range is sufficient to measure SREM_B_ and gVLM^23^ in the majority of participants.

### Dietary precursors of SREM_B_

The importance of the specificity of nutritional biomarkers has been discussed previously^5,19^, but in contrast to the relationship between nutritional biomarker and intake, this is not often assessed. We have shown that (−)-epicatechin, but none of the other flavan-3-ols tested, resulted in the formation of SREM_B_. This confirms results from our previous studies^3,25^, showing that oligo- and polymeric procyanidins are not cleaved into their flavan-3-ol subunits.

The data therefore show that urinary SREM_B_ is specific for (−)-epicatechin, but considering that this compound could be present in its enantiomeric form (+)-epicatechin, it is worth questioning whether SREM_B_ could be specific to assess (−)-epicatechin intake. In this context, the biosynthetic pathways of flavonoids in plants result predominantly in (−)-epicatechin^26^ and only small amounts of (+)-epicatechin are found in the diet^27^ and thus not relevant as a contributor to SREM_B_. Furthermore, previous data show that (+)-epicatechin intake does not significantly contribute to epicatechin-3′-β-glucuronide, one of the main contributors to the set of metabolites included in SREM_B_^28^. Thus, SREM_B_ will predominantly reflect the intake of (−)-epicatechin.

### Association between dietary intake and SREM_B_

We have shown a statistically significant linear association between (−)-epicatechin and SREM_B_ excretion in 24 h urine in an intake-escalation study (R^2^: 0.86 (95% CI 0.8l; 0.92)). Importantly, the association of individual metabolites showed a weaker association with intake than did the sum (SREM_B_). We demonstrated that this is due to changes in the relative contribution of different SREM_B_ metabolites according to intake amount. This observation is not novel and concurs with previous studies demonstrating a change in sulfation/glucuronidation ratio with the amount of phenolic compounds ingested^29^. Thus, these results show that it is important to use a combination of different metabolites as biomarkers of intake, as changes in metabolism due to intake could result in mis-estimations of intake. The combination of metabolites permits both, improving the strength of the association and increasing the recovery assessed for (−)-epicatechin. Overall, the data presented here show that changes in urinary excretion of SREM_B_ reflect changes in dietary intake of (−)-epicatechin consistently and are therefore prognostic of intake.

### Suitability of SREM_B_ as nutritional biomarker of (−)-epicatechin intake

Our results show that 24 h urinary SREM_B_ excretion meets the criteria set to evaluate candidate nutritional biomarkers. Furthermore and according to the criterion for biomarker evaluation used previously by the NPAAS-FS (Nutrition and Physical Activity Assessment Study Feeding Study; R^2^ ≥ 0.36)^30,31^, the data also suggest that 24 h excretion of SREM_B_ can be used as recovery biomarker of (−)-epicatechin and therefore used to estimate actual (−)-epicatechin intake. The correlation between intake and biomarker is >0.8 and therefore meets the criteria commonly used for recovery biomarkers^5^. Furthermore, the data available from this and previous studies, in particular the pharmacokinetic data^32^, provide precise and quantitative knowledge of the physiological balance between intake and output which is required for recovery biomarkers^33^. In comparison with urinary nitrogen as biomarker of dietary protein intake, one of the best characterized recovery biomarkers, the correlations between intake and biomarker are comparable (0.8–0.9 for nitrogen as a biomarker of dietary protein^34^, 0.9 for urinary SREM_B_ as biomarker of epicatechin intake). Although the recovery of (−)-epicatechin as urinary SREM_B_ (10% ± 1%) was lower than the recovery of dietary nitrogen in urine (91% ± 1%^34^), the variability of recovery was similar and most notably the recovery was neither skewed nor dependent on intake. When applying this biomarker to the 24 h urine samples collected in *Challenge*, the estimated intake of (−)-epicatechin was 14.0 ± 1 mg/d, ranging from 0 to 58 mg/d.

### Comparison with γ-valerolactone metabolites (gVLM)

We have previously evaluated gVLM as biomarker of flavan-3-ol intake and shown that, due to its plasma half-life of approximately 6 h^32^ and the frequent consumption of flavan-3-ol containing foods, it can be used as surrogate^35^ or concentration biomarker both in 24 h urine and spot urine samples^6^. However, the large inter-individual variability in colonic microbial metabolism makes gVLM not suitable as recovery biomarker. Conversely, the short plasma half-life of SREM_B_, approximately 2 h, assures a complete excretion within 24 h of urine collection that, added up to the high association with intake, makes SREM_B_ ideal for a recovery biomarker. Due to the different flavanols contributing to gVLM and SREM_B_ and differences in half-life, the measurement of both biomarkers could provide complementary information. In 24 h urine samples, a combination of SREM_B_ and gVLM could be used to differentiate between (−)-epicatechin and total flavan-3-ol intake, whereas in spot urine samples, SREM_B_ can be used as marker of very short-term, acute (−)-epicatechin intake (see Table 4 for a direct comparison).

**Table 4:** Comparison of gVLM and SREM_B_ as biomarkers.

	gVLM	SREM_B_
Sample	24 h urine, spot urine	24 h urine
Metabolites included	gVL3′S, gVL3′G, gVL4′G	E3′S, E3′G, 3′Me5S
Specificity	Flavan-3-ol monomers, ECG, procyanidins	(−)-epicatechin
Type of biomarker	Concentration/surrogate	recovery

### Strengths and limitations

This study has a number of strengths, but also some limitations which need to be considered when interpreting results. A key strength of the study is the carefully validated analytical method and the availability of authentic standards for all three (–)-epicatechin metabolites, and we have shown previously that this is a major contributor to the accuracy of polyphenol metabolite quantification by LC-MS^23^. The reliance on a single deuterium-labelled internal standard (D_2_/D_3_-E3′G) for quantification of E3′G, E3′S and 3′Me5S might have affected quantification of the latter two metabolites, but the method validation showed very good precision and accuracy. A further strength of the study is the careful investigation of the intake response relationship and in particular the specificity of SREM_B_. Providing the flavan-3-ol intervention on a single occasion using a defined test material was different from what could be expected as part of the habitual diet, but permitted a tight control on the amounts and type of flavan-3-ols consumed during the intervention and thus, obtain essential information on the specificity and intake-amount relationship of SREM_B_ as biomarkers of (–)-epicatechin intake. A limitation of the study is the reliance on two UK cohorts to evaluate the applicability of SREM_B_ as urinary biomarker. While flavan-3-ol intake in the UK is generally higher than in many other countries, their main source is tea^10,18^ with a very different flavan-3-ol composition than for example pome fruits. While this ensured that there was a wide range of flavanol intake within the study population, it could have affected outcomes. It is therefore important to apply this biomarker to studies from countries with more diverse sources of flavanols. Finally, it should be noted that the criteria chosen for the evaluation of SREM_B_ have not been specifically created for nutritional biomarkers of dietary bioactives. The criteria used were derived from the frameworks provided by IARC and IOM. Future discussions, such as suggested previously^36^, are crucial to develop specific criteria for the evaluation of nutritional biomarkers, and thus foster the development of new biomarkers of dietary bioactives for the replacement of biased methods for intake assessment.

## Conclusion

In our studies, we have shown that 24 h urinary excretion of a combination of three structurally related (−)-epicatechin metabolites ((−)-epicatechin-3′-glucuronide (E3′G), (−)-epicatechin-3′-sulfate (E3′S) and 3′-O-methyl-(−)-epicatechin-5-sulfate (MeE5S), SREM_B_) meet the criteria for a recovery biomarker in 24 h urines and can therefore be used as a nutritional biomarker to estimate actual (−)-epicatechin intake. The method we have developed is suitable for automation and therefore high-throughput analysis, and we have applied it successfully to more than 24,000 samples of the EPIC Norfolk cohort. The development of this biomarker would not have been possible without the availability of authentic standards and detailed pharmacokinetic data.

We have shown that SREM_B_ is specific for (−)-epicatechin and not any other flavan-3-ol commonly consumed. Our findings provide an accurate method for the objective estimation of dietary (−)-epicatechin, the most likely bioactive flavan-3-ol. This opens up the possibility to investigate associations between (−)-epicatechin intake and health outcomes in large-scale observational studies. In the future this method could hold the potential to objectively assess dietary epicatechin adequacy as part of approaches for personalised nutrition goals.

## Supplementary information

Supplementary material
