# Supplementary material for: Evaluation of (−)-epicatechin metabolites as recovery biomarker of dietary flavan-3-ol intake
Source: Sci Rep. 2019 Sep 11;9:13108. doi: 10.1038/s41598-019-49702-z (PMC6739331; doi:10.1038/s41598-019-49702-z)
Supplement: Supplementary file 1 — Supplementary material [file 41598_2019_49702_MOESM1_ESM.pdf]

Evaluation of (–)-epicatechin metabolites as recovery biomarker of dietary flavan-3-ol intake

Javier I. Ottaviani[1], Reedmond Fong[2], Jennifer Kimball[2], Jodi L. Ensunsa[2], Nicola Gray[3], Anna Vogiatzoglou[4], Abigail Britten[5], Debora Lucarelli[5], Robert Luben[6], Philip B. Grace[7], Deborah H. Mawson[7], Amy Tym[7], Antonia Wierzbicki[7], A. David Smith[8], Nicholas J. Wareham[5], Nita G. Forouhi[5], Kay Khaw[6], Hagen Schroeter[1], Gunter G. C. Kuhnle[3,6,*]

[1]Mars, Inc., McLean, VA; [2]Department of Nutrition, UC Davis, CA; [3]Department of Food and Nutritional Sciences, University of Reading, UK; [4]School of Life Sciences, Coventry University, UK; [5]MRC Epidemiology Unit, University of Cambridge, UK; [6]Department of Public Health and Primary Care, University of Cambridge, UK; [7] LGC, Newmarket Road, Fordham, UK; [8]OPTIMA Department of Pharmacology, University of Oxford, UK

*Corresponding author: Gunter G. C. Kuhnle; Department of Food & Nutritional Sciences, University of Reading, Reading RG6 6UR, UK. Telephone: +44 118 378 7723; Email: g.g.kuhnle@reading.ac.uk

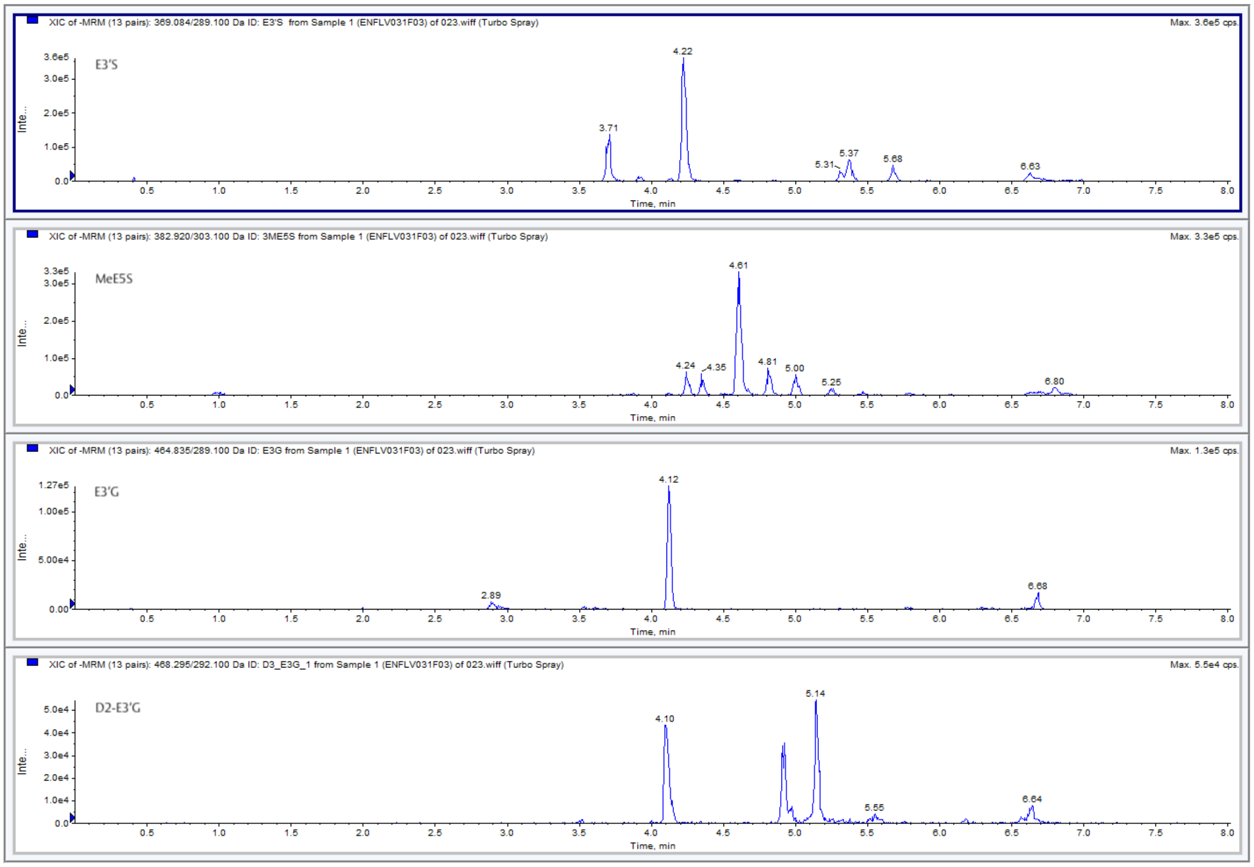

Supplemental Figure 1: Typical chromatogram of a spot urine sample of EPIC Norfolk.

(−)-epicatechin-3′-β-D-glucuronide (E3’G)

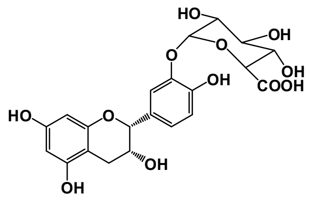

(−)-epicatechin-3′-sulfate (E3’S)

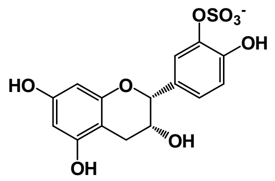

3′-O-methyl(−)-epicatechin-5-sulfate 3ME5S

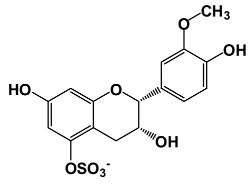

D_2/D_3 epicatechin-3’-β-D-glucuronide; 50:50 mix (D_2/D_3E3’G)

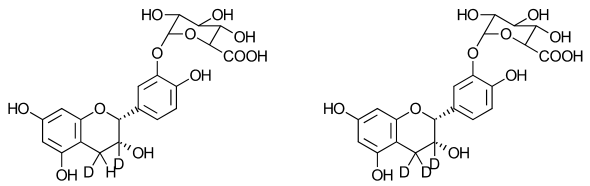

Supplemental Figure 2: Structures of $SREM_B$ (top row) and internal standard mix (bottom row)

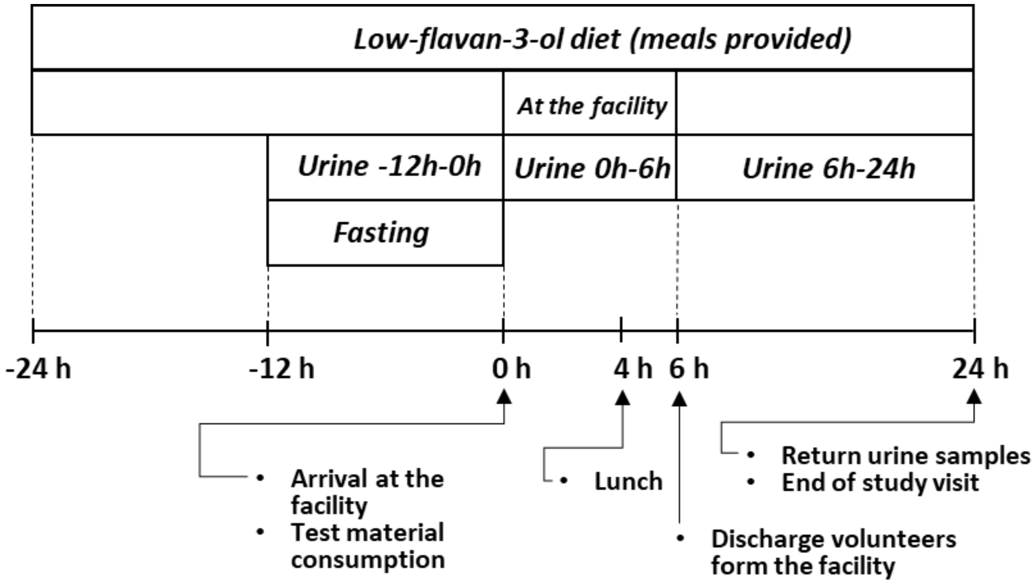

Supplemental Figure 3: Scheme depicting the different events and steps involved in each study visit of the dietary intervention studies conducted, including Specificity Study, Intake escalation Study and Inter-individual variability study. These events were repeated 8 times, 4 times and 2 times for the Specificity Study, Intake escalation Study and Inter-individual variability study, respectively. Study visits were scheduled once a week, but never than 3 days apart.

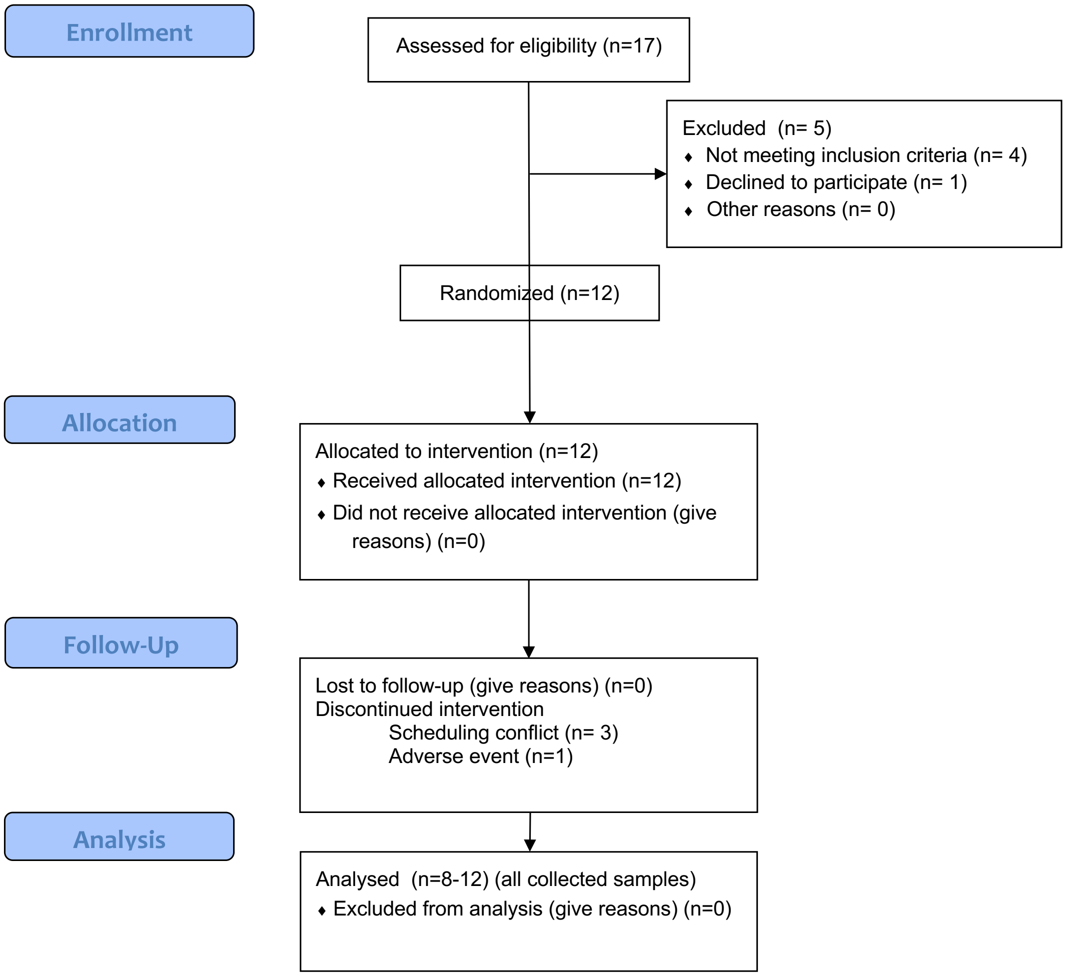

Supplemental Figure 4: Participant flow chart for the specificity study.

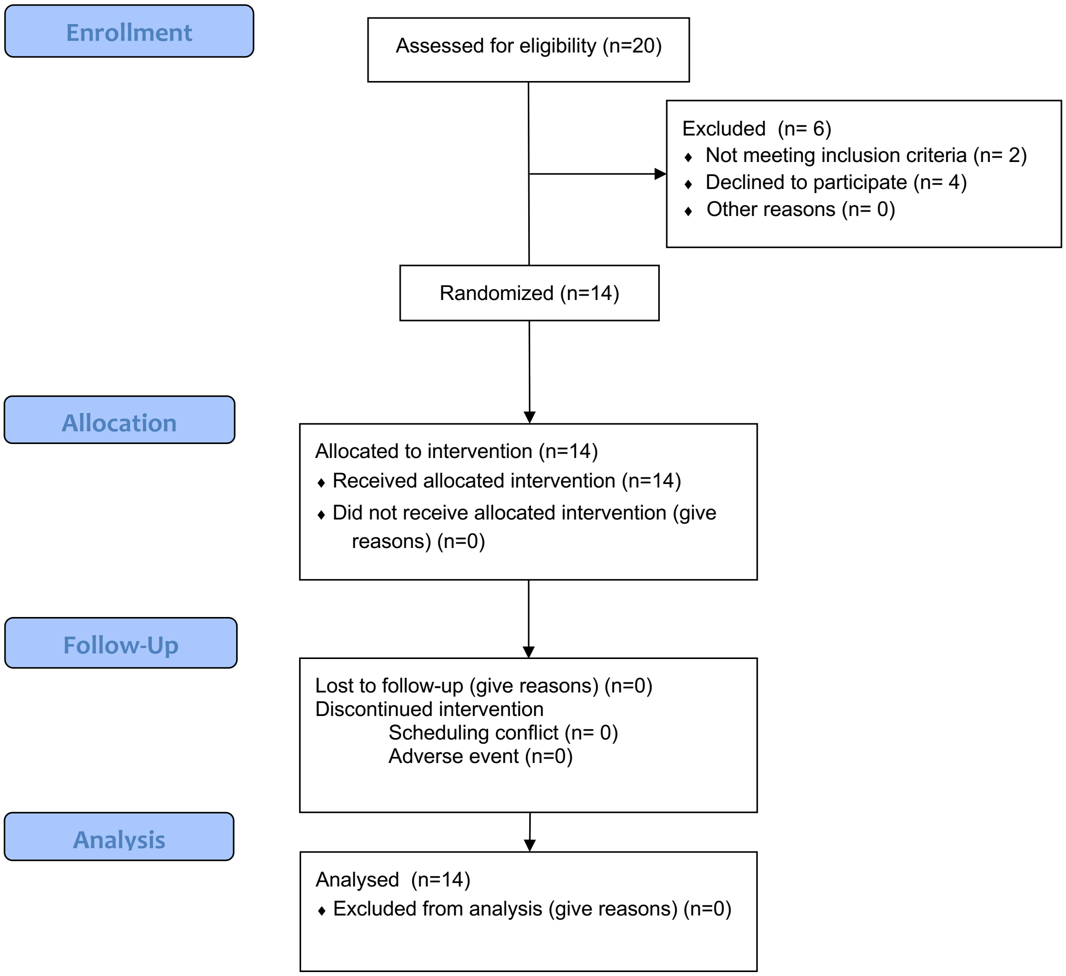

Supplemental Figure 5: Participant flow chart for the intake-response study.

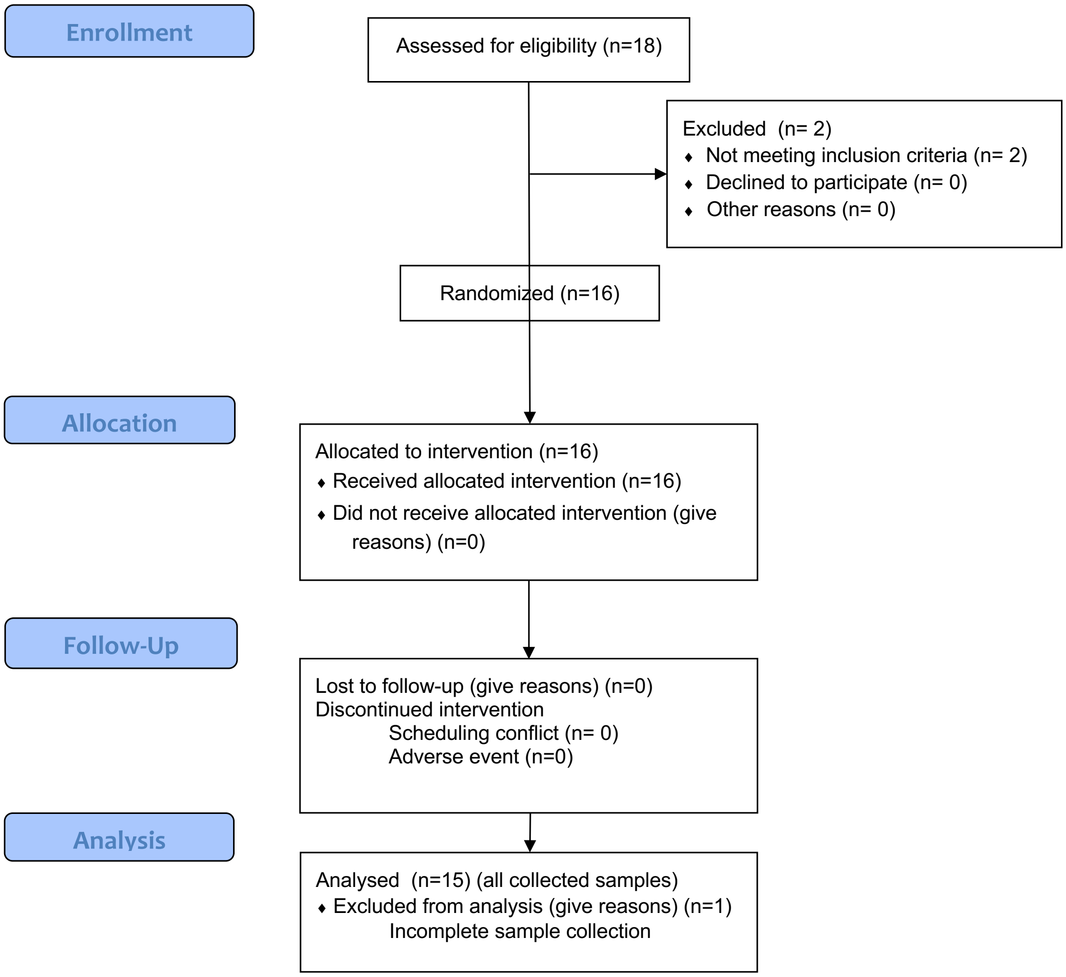

Supplemental Figure 6: Participant flow chart for the intra-individual variability study.
